# Whole-Cell and Acellular Pertussis Vaccines: A Narrative Review of Biological, Clinical, Safety and Programmatic Evidence, with Implications for Poland

**DOI:** 10.3390/vaccines14090775

**Published:** 2026-09-03

**Authors:** Andrzej Fal, Iwona Paradowska-Stankiewicz, Aneta Nitsch-Osuch, Ernest Kuchar

**Affiliations:** 1 Collegium Medicum, Cardinal Stefan Wyszynski University, 01-938 Warsaw, Poland; 2 Department of Infectious Disease Epidemiology and Surveillance, National Institute of Public Health NIH—National Research Institute, Chocimska 24, 00-791 Warsaw, Poland; 3 Department of Social Medicine and Public Health, Medical University of Warsaw, Pawinskiego 3a, 02-106 Warsaw, Poland; 4 Department of Pediatrics with Clinical Assessment Unit, Medical University of Warsaw, Zwirki i Wigury 63A, 02-091 Warsaw, Poland

**Keywords:** whooping cough, *Bordetella pertussis*, immunologic memory, immunity, mucosal, disease outbreaks, vaccination hesitancy, health policy

## Abstract

**Background/Objectives:** Pertussis has resurged worldwide despite long-standing vaccination programs. Whole-cell (wP) and acellular (aP) pertussis vaccines differ in immunobiology, durability, reactogenicity, and programmatic use. Poland is the only European Union/European Economic Area (EU/EEA) country that routinely primes infants with wP while using aP products for boosters, pregnancy, and selected indications, providing an informative programmatic context. This review compares both platforms across biological, clinical, safety, epidemiological, and supply domains. **Methods:** We conducted a narrative review of PubMed/MEDLINE literature and documents from the World Health Organization, European Centre for Disease Prevention and Control, national public health institutes, and regulatory authorities through July 2026. No protocol was registered and no quantitative synthesis was performed. **Results:** wP priming induces a persistent T-helper 1/T-helper 17-oriented response and an immunoglobulin G1-dominant profile, whereas aP vaccination produces higher, higher-avidity antibody concentrations, an increasing immunoglobulin G4 fraction, and substantially lower reactogenicity. Historical trials demonstrated marked product-level heterogeneity: efficacy ranged from 36–48% for poorly performing wP vaccines to 84–85% for multicomponent aP vaccines, while one United Kingdom wP product performed comparably to a five-component aP vaccine. Protection after aP schedules wanes within several years, no validated correlate of protection exists, and neither intramuscular platform reliably prevents colonization or transmission. In 2024, eight aP-using EU/EEA countries had higher pertussis notification rates than Poland despite stable Polish infant coverage. **Conclusions:** Neither platform is uniformly superior across products, schedules, and clinically relevant outcomes. Program performance depends primarily on timely infant vaccination, maternal vaccination, appropriate boosters, product-specific effectiveness evidence, and secure vaccine supply.

## 1. Introduction

Pertussis remains endemic worldwide and has resurged in many countries with long-standing, high-performing childhood vaccination programs. Contemporary analyses attribute this rebound to a set of factors rather than to a single cause: waning post-vaccination immunity, accumulation of susceptible adolescents and adults, more sensitive molecular diagnostics, intensified surveillance and case ascertainment, pathogen adaptation and, after the COVID-19 pandemic, release of accumulated susceptibility following prolonged non-pharmaceutical control measures [1,2]. *Bordetella pertussis* remains highly transmissible and continues to circulate in settings with high infant series completion, so a rise in reported incidence should not be taken as evidence that a particular vaccine platform has failed [2,3].

Two vaccine platforms are in routine use. Whole-cell vaccines (wP) contain killed whole *B. pertussis* organisms and therefore present a broad array of bacterial antigens together with intrinsic innate stimuli. Acellular vaccines (aP) contain a defined set of purified antigens: pertussis toxin alone or combined with filamentous hemagglutinin, pertactin and fimbrial agglutinogens, adsorbed to an aluminum adjuvant. Because pertussis antigens are almost always supplied in combination with diphtheria and tetanus toxoids, the abbreviations used throughout this review follow international convention: DTwP and DTaP denote pediatric combinations containing full-dose diphtheria and tetanus toxoids with whole-cell or acellular pertussis components, respectively, whereas Tdap denotes a booster formulation with reduced-antigen-content diphtheria toxoid and reduced-antigen-content acellular pertussis components, licensed for older children, adolescents, adults and pregnant women. Tdap is not an infant-priming product; it exists because full-dose diphtheria toxoid becomes progressively more reactogenic with repeated administration, and it is the formulation on which almost all adolescent, adult, and maternal pertussis vaccination worldwide now depends.

The World Health Organization (WHO) advises that national programs currently using wP vaccination should continue to use wP for the primary series, and that a change from wP to aP should be considered only where additional periodic boosters or maternal immunization can be assured and sustained [4]. This position expresses a judgment about program performance under real-world constraints rather than a claim that one platform is biologically superior in all respects.

Poland occupies an unusual position in Europe. According to ECDC surveillance and the ECDC Vaccine Scheduler, Poland is the only EU/EEA country that uses a wP-containing vaccine for routine infant priming in the general population [5,6]. Acellular products are used in Poland for subsequent booster doses, for vaccination in pregnancy, for infants with contraindications to wP and for infants born before 37 weeks of gestation or with a birth weight below 2500 g, and are also available as privately purchased combination vaccines [6,7]. Both platforms therefore operate in parallel within a single healthcare system and population, creating an opportunity to examine their complementary roles under broadly comparable epidemiological and organizational conditions. The ongoing national debate about a possible transition from wP-based priming to an aP-only schedule is relevant beyond Poland, as it raises general questions about the durability of protection, reactogenicity, vaccine confidence, program cost, supply security, and the feasibility of sustaining booster and maternal coverage after such a transition.

This review has three objectives: to synthesize the biological, clinical, safety, epidemiological and programmatic evidence comparing wP and aP vaccines; to distinguish where that evidence is strong from where it is inconclusive; and to derive proportionate consequences for the Polish immunization program, including an examination of what the 2021–2024 Polish resurgence can and cannot be attributed to. The review does not advocate for either platform.

## 2. Scope and Approach

This is a narrative review. Literature was identified through searches of PubMed/MEDLINE and through the reference lists of retrieved articles, complemented by official documents from WHO, ECDC, national public health institutes and regulatory authorities. Priority was given to placebo-controlled and randomized efficacy trials, systematic reviews, large observational effectiveness studies, mechanistic human immunology studies, official position papers and national surveillance reports available up to July 2026. No protocol was registered, no systematic search strategy was applied, and no formal risk-of-bias assessment or quantitative synthesis was undertaken; the review is reported as a narrative synthesis and does not claim compliance with PRISMA or any other systematic-review reporting guideline. The selection of sources was purposive and reflects the authors’ judgment of relevance to the platform comparison and the Polish programmatic context.

Product-level performance matters throughout. “wP” does not denote a single standardized vaccine: strain selection, number of organisms per dose, cultivation conditions, inactivation and detoxification methods, formulation and potency control all differ between manufacturers [8,9]. Acellular vaccines likewise differ in the number, quantity, detoxification and presentation of their pertussis antigens. Program comparisons that classify countries only as “wP” or “aP” can therefore conceal clinically relevant variation in products, dose timing, booster policy, maternal vaccination, series completion, surveillance intensity and diagnostic practice [3].

## 3. Global Pattern of Platform Use

Global immunization performance is usually monitored by coverage with three doses of the diphtheria–tetanus–pertussis (DTP) vaccine. In 2025, approximately 85% of infants worldwide (about 110 million children) received three DTP-containing doses, while 13.5 million children received no doses at all [10]. WHO recommends that countries currently using wP continue to do so for the primary series, and most low- and middle-income countries, where the great majority of pertussis deaths occur, use wP-containing combination products for infant priming [4]. High-income countries have largely transitioned to aP-containing combinations, and aP is the only platform used anywhere for boosters in older children, adolescents, adults and pregnant women [3,4]. Within the EU/EEA, all countries include pertussis vaccination in the routine childhood schedule and all except Poland use aP-containing vaccines for primary immunization [5]. Any European debate about the relative merits of the two platforms, therefore, has, in effect, a single contemporary EU/EEA reference program.

## 4. Comparative Immunobiology

### 4.1. Antigen Breadth and T-Helper Polarization

Whole-cell vaccines expose the immune system to a broad array of bacterial antigens and innate immune stimuli, whereas acellular vaccines contain a defined combination of purified antigens. This compositional difference shapes innate activation and the subsequent adaptive response [11].

Human studies consistently show that wP priming promotes a T helper 1 (Th1)/T helper 17 (Th17)-oriented cellular response, whereas alum-adjuvanted aP vaccination tends to elicit a more Th2-skewed profile [11]. Th1- and Th17-associated responses contribute to interferon-γ and interleukin-17 production, macrophage activation, neutrophil recruitment, and bacterial clearance at respiratory surfaces [12]. The immune phenotype established in infancy can persist into adolescence despite repeated aP boosters, so that the cellular response profile of an adolescent is largely determined by the vaccine type received in the first year of life [12,13]. This phenomenon is commonly described as immunological imprinting.

Polarization should nevertheless not be treated as a validated correlate of clinical protection. No single serological or cellular marker has been shown to predict protection against pertussis across products and populations. Differences in cytokine profiles, antibody subclasses, or memory-cell phenotypes, therefore, cannot by themselves justify a change in platform or establish clinical superiority.

### 4.2. Persistence of Cellular and Humoral Memory After Whole-Cell and Acellular Priming

Direct comparisons of the two platforms in the same cohorts are the most informative source on memory persistence, and they show that each platform leaves a distinct signature rather than one being uniformly stronger.

Studies of wP-primed individuals indicate a durable cellular signature. Adults primed with wP in childhood retain respiratory tissue-resident memory CD4 T cells capable of producing interleukin-17 and interferon-γ decades later [14]. This supports the biological plausibility of persistent imprinting but does not demonstrate decades-long sterilizing immunity or sustained clinical effectiveness. Tissue-sampling studies are mechanistic in design and typically involve small, selected groups of participants; their findings should not be extrapolated directly to population incidence [3,14].

Acellular priming has been characterized in the same Dutch cohorts and yields a complementary picture. Two years after a preschool aP booster, children primed in infancy with aP had higher pertussis-specific antibody concentrations and higher antibody avidity indices than wP-primed children, and memory B cells remained detectable in both groups; the antibody response of aP-primed children, however, contained a larger immunoglobulin G4 (IgG4) fraction, and their T-cell responses were less Th1-oriented than those of wP-primed children [15]. Following the same children into adolescence confirmed that the priming platform, rather than the booster, determined the cellular profile [13]. Taken together, these paired observations show that aP priming yields quantitatively higher and higher-avidity antibody responses that are more readily boosted. In contrast, wP priming leaves a more Th1/Th17-oriented cellular signature that remains detectable into adolescence. Neither pattern alone identifies the platform that protects better, which is why the clinical endpoints in Section 5 are decisive.

### 4.3. Humoral Response Quality and Its Relationship to Protection

Acellular and whole-cell priming generate different antibody-subclass profiles. Repeated aP exposure is associated with a progressively greater IgG4 fraction, whereas IgG1-dominant responses predominate after wP priming or natural infection [11,15]. In a Dutch longitudinal comparison, both the proportion and the concentration of antigen-specific IgG4 at 4 years of age were significantly higher in aP-primed than in wP-primed children, and remained higher after two successive aP boosters at 4 and 9 years; the shift extended to the co-administered diphtheria and tetanus antigens, indicating that the priming platform sets the subclass profile of the whole combination response [16].

The functional relevance of this shift follows from the effector properties of the two subclasses. IgG1 fixes complement and engages Fcγ receptors efficiently, supporting opsonophagocytosis and complement-mediated killing, whereas IgG4 does not activate complement and binds Fcγ receptors weakly; a rising IgG4 fraction therefore reduces the effector capacity delivered per unit of total antigen-specific IgG, and the investigators who described the shift proposed that it may contribute to reduced protection in some aP-primed children [11,16].

This mechanism is plausible but must be kept distinct from a demonstrated correlate. The closest available evidence comes from the Swedish efficacy trials, in which pre-exposure serum was linked to subsequent clinical outcome after household exposure: concentrations of IgG against pertactin, fimbriae 2/3 and pertussis toxin were significantly associated with clinical protection, and the vaccines of highest measured efficacy induced higher anti-pertactin and anti-fimbriae responses than less efficacious products [17]. Antibody specificity and quantity are thus demonstrably related to protection. In contrast, the subclass distribution associated with a given antibody concentration has not been shown to predict clinical outcome, and no threshold titer defines an individual as protected [11,17]. Two practical consequences follow. First, quantitative serological comparisons between platforms cannot be converted into statements about clinical protection. Second, the IgG4 shift is a legitimate reason to investigate the durability of aP-based schedules, but not in itself a reason to prefer or reject either platform.

Functional differences have also been observed in randomized comparisons of the platforms themselves. In Gambian infants born to mothers who received tetanus toxoid only, a whole-cell primary series produced a higher pertussis toxin-specific IgG geometric mean concentration at 9 months than an acellular primary series (adjusted geometric mean ratio, 2.02; 98.75% confidence interval [CI], 1.15–3.55) [18].

### 4.4. Antigenic Adaptation Under Vaccine Pressure

Acellular vaccines focus immune pressure on a limited set of antigens, and the global expansion of pertactin-deficient *B. pertussis* lineages is an important example of vaccine-associated pathogen adaptation [19,20]. In United States surveillance of isolates collected between 2011 and 2013, 640 of 753 isolates (85%) did not express pertactin; case-patients who had received at least one pertussis vaccine dose had higher odds of infection with a pertactin-negative strain than unvaccinated case-patients (adjusted odds ratio 2.2, 95% CI 1.3–4.0) [21]. Whole-cell vaccines, which present a wider antigenic repertoire, may exert less such selective pressure. The public health implications of these data are nuanced: loss of pertactin does not necessarily abolish vaccine protection, and the contribution of pertactin deficiency to resurgence varies across settings [3,20]. Reported national proportions of pertactin-negative isolates depend on sampling period, laboratory participation and case ascertainment, and should not be treated as fixed values.

### 4.5. Mucosal Protection, Colonization and Transmission

Neither platform, administered intramuscularly, reliably induces sterilizing mucosal immunity. In a non-human primate model, aP vaccination protected against clinical disease but did not prevent nasopharyngeal colonization or onward transmission, whereas prior infection did [22]. Whole-cell vaccination shortened the duration of colonization in that model but likewise did not prevent it. Extrapolation from animal models to human transmission dynamics is uncertain, and there is no direct human evidence establishing a decisive transmission advantage for either platform. The clinical corollary is that immediate protection of very young infants depends primarily on maternal vaccination and on timely, complete infant priming, rather than on interrupting community transmission by either vaccine class.

This limitation is the explicit rationale for mucosal candidates now in clinical development. BPZE1, a live attenuated intranasal vaccine, induced mucosal and systemic anti-pertussis immune responses in a randomized phase 2b comparison with Tdap in adults [23], and in a subsequent placebo-controlled phase 2b trial using a controlled human infection model, 12 of 20 participants (60%, 95% CI 39–78) in the BPZE1 group versus 4 of 16 (25%, 95% CI 10–50) in the placebo group had no detectable colonization with *B. pertussis* on days 9, 11 and 14 after challenge in the per-protocol adequate-inoculum population (*p* = 0.033); the corresponding difference in the modified intention-to-treat population was not statistically significant (58%, 95% CI 39–76 versus 33%, 95% CI 17–55; *p* = 0.091) [24]. These are small early-phase trials in adults that use a challenge endpoint rather than clinical disease, and they do not support any change in current practice; they indicate that the colonization gap common to both licensed platforms is being addressed by a distinct technology rather than by choosing between wP and aP.

## 5. Efficacy, Effectiveness and Durability

### 5.1. Heterogeneity of Whole-Cell Products and Its Origins

Historical placebo-controlled trials of the 1990s demonstrate substantial product-level heterogeneity within both platforms. In the Italian trial, each of two three-component aP vaccines showed an efficacy of 84% (95% CI 76–88) against typical pertussis, compared with 36% (95% CI 14–52) for the wP comparator [25]. In the contemporaneous Swedish trial, efficacy against pertussis linked to a laboratory-confirmed case or to household contact with a prolonged cough was 85.2% (95% CI 80.6–88.8) for a five-component aP vaccine, 58.9% (95% CI 50.9–65.9) for a two-component aP vaccine and 48.3% (95% CI 37.0–57.6) for a United States-licensed wP vaccine [26]. In a subsequent Swedish trial of 82,892 infants that used a United Kingdom wP vaccine as comparator, the whole-cell product performed at least as well as the five-component aP vaccine and significantly better than a three-component aP vaccine against typical pertussis in intention-to-treat analysis [27]. In a randomized trial in Senegal, a two-component aP vaccine was less protective than the wP comparator, with a pertussis incidence ratio of 1.54 (95% CI 1.23–1.93) [28].

The Cochrane review of acellular vaccines in children summarized this pattern, estimating the efficacy of multicomponent (three or more antigens) aP vaccines at 84–85% against typical whooping cough and 71–78% against mild disease, with lower estimates of 59–78% for one- and two-component products, and concluding that multicomponent aP vaccines are more efficacious than low-efficacy wP vaccines but may be less efficacious than the highest-efficacy wP vaccines [29].

The phrases “low-efficacy” and “highest-efficacy” whole-cell vaccines therefore refer to specific licensed products, not to two subtypes of a defined technology, and manufacturing differences are an important contributor to this heterogeneity. Whole-cell vaccines are suspensions of inactivated bacteria, and their protective content depends on the *B. pertussis* strain or strain combination used for production and its match to circulating strains, the number of organisms per dose, the cultivation conditions that determine expression of protective antigens such as pertussis toxin, pertactin and fimbriae, and the inactivation and detoxification method, which can preserve or destroy protective epitopes [8,9]. Because no *in vitro* correlate of protection was available, batch release depended on the mouse intracerebral challenge assay, which measures protection against intracerebral infection rather than the antigen composition relevant to human respiratory disease; its relationship to clinical efficacy is indirect, and the resulting quality control tolerated substantial variation in protective content between products [9]. Immunological data are consistent with a contribution from manufacturing and antigen composition: the wP and aP vaccines with the highest measured efficacy in the Swedish trials were those that induced the highest anti-pertactin and anti-fimbrial responses [17]. Estimated efficacy is also sensitive to study design, case definition, and age distribution, which accounted for part of the historical variation among wP field studies [8].

These figures should therefore be read as product-specific rather than platform-defining. A poorly performing wP product does not characterize the platform, and a high-performing multicomponent aP product does not characterize all acellular vaccines. Comparative statements at the level of “wP versus aP” are of limited value for policy unless the specific products, schedules and settings are stated [3,30]. The corollary for any country considering a platform change is that the relevant question is not which platform to adopt but which product, on which schedule, with what evidence of effectiveness.

### 5.2. Durability of Protection

Protection after aP priming and boosting wanes within a few years. In a Kaiser Permanente case–control study, the odds of acquiring pertussis increased by an average of 42% per year after the fifth DTaP dose (odds ratio 1.42, 95% CI 1.21–1.66 versus polymerase chain reaction [PCR]-negative controls) [31]. In the 2012 Washington State epidemic, the effectiveness of adolescent Tdap was 73% (95% CI 60–82) within the first year after vaccination and 34% (95% CI −0.03 to 58) at two to four years; the confidence interval for the later estimate includes no effect, so residual protection at that interval cannot be regarded as established [32].

The corresponding comparison between platforms within the same population points in the same direction. Among Kaiser Permanente teenagers born in 1994–1999 who had received four pertussis-containing doses in the first two years of life, those who had received four DTaP doses were considerably more likely to test PCR-positive during the 2010–2011 outbreak than those who had received four DTwP doses (odds ratio 5.63, 95% CI 2.55–12.46), with an intermediate risk for mixed schedules (odds ratio 3.77, 95% CI 1.57–9.07) and a significant dose–response relationship with the number of DTwP doses received [33]. Epidemiological reviews of the United States and Australian epidemics reached a concordant conclusion: namely, that the cohorts primed exclusively with aP experienced the highest adolescent attack rates, and that receipt of wP, at least as the first dose, was associated with greater and longer-lasting protection [34]. These are observational comparisons of historical products, subject to confounding by birth cohort, calendar time, and diagnostic practice, and they cannot be read as estimates of what a contemporary wP product would achieve today.

A Polish seroepidemiological study of 2745 children and adolescents provided a useful concurrent comparison of the two priming platforms. Among participants, 1161 (42.5%) had received wP, and 1314 (48.1%) had received aP for the primary series and the toddler booster (percentages as published; the two priming groups do not account for the full sample of 2745). Vaccination timeliness was suboptimal: 53.57% received doses 2–4 at the recommended intervals, and 41.52% received all five doses on time. In both priming groups, anti-pertussis-toxin antibody concentrations showed a re-increase consistent with recent infection from approximately 8 years after the school-entry booster, with no significant difference between wP- and aP-primed participants in the timing or intensity of this re-increase [35]. The authors therefore concluded that the timing and intensity of waning appeared similar after wP and aP priming. These are serological, not clinical, endpoints, and, in the absence of a validated correlate of protection, they should not be equated directly with the duration of clinical protection.

WHO notes that in countries using wP, protection can be expected to last for several years, and this expectation underlies the advice that wP-using programs continue with wP for the primary series unless sustained boosters or maternal immunization can be assured [4].

### 5.3. Mixed and Modeled Schedules

Mixed schedules have been evaluated as a means of retaining the immunological features of wP priming while limiting reactogenicity. In the Australian OPTIMUM randomized, double-blind non-inferiority trial, a mixed schedule beginning with a whole-cell first dose met the prespecified non-inferiority criterion for pertussis toxin-specific IgG at 6 months, with a geometric mean ratio of 0.98 (95% CI 0.77–1.26) relative to an all-acellular schedule [36]. Modeling studies have suggested that wP priming could reduce pertussis incidence and associated costs in some settings [37]; such analyses depend on assumptions about waning, transmission, and reporting and are hypothesis-generating rather than confirmatory. Trials and models of this kind identify plausible options but do not by themselves establish a clinical benefit that would justify a change in national policy.

## 6. Reactogenicity and Safety

The difference in reactogenicity between the platforms is one of the most consistent findings in the field and is clinically important. Whole-cell vaccines are associated with substantially higher rates of fever, injection-site reactions, irritability, and persistent crying than acellular vaccines. A systematic review of pertussis vaccine safety reported a markedly higher risk of fever after wP than after aP vaccination (relative risk, 9.21; 95% CI, 5.39–15.76), with correspondingly higher risks of local reactions [38]. The Cochrane review reached the same qualitative conclusion for both the primary series and booster doses [29], and the Swedish placebo-controlled trial found significantly higher rates of protracted crying, cyanosis, fever and local reactions with the wP vaccine. In contrast, the aP vaccines did not differ appreciably from the diphtheria–tetanus control [26].

Reactogenicity should not be confused with serious vaccine-related harm. The historical controversy over whole-cell pertussis vaccines and permanent neurological injury was examined by the United States Institute of Medicine, which concluded that the evidence did not establish a causal relationship between the whole-cell pertussis vaccine and permanent brain damage [39]. Subsequent work reinforced this conclusion: several children with encephalopathy temporally associated with wP vaccination were later found to carry *SCN1A* mutations that cause Dravet syndrome, indicating that vaccination served as a trigger for the first manifestation of a genetically determined encephalopathy rather than its cause [40]. Large active-surveillance programs have not identified an excess of serious neurological outcomes; Canadian active surveillance covering more than 6.5 million doses did not detect an increased risk of such events after pertussis vaccination [41]. Long-term Japanese experience with acellular pertussis vaccination also provides supportive safety data for aP products [42].

Two pragmatic implications follow. First, higher reactogenicity is a real burden for children and families, affects acceptance as well as adherence to the schedule, and is a legitimate consideration in program design. Second, higher reactogenicity does not indicate a higher risk of serious harm, and conflating the two in communication is inaccurate and counterproductive. The key features of whole-cell (wP) and acellular (aP) pertussis vaccines are summarized in Table 1. 

## 7. Maternal Vaccination and Infant Priming

Young infants have the highest risk of hospitalization and death from pertussis before the primary series is complete. In the EU/EEA in 2024, infants under one year of age accounted for 41% of hospitalized cases with known hospitalization status, and 44 of 84 reported deaths occurred in infants aged one year and under. The severity gradient is steeper than these proportions alone convey: among cases in infants aged one year and under with known hospitalization status, 61% were hospitalized, compared with 7.5% of all reported cases, and this age group had the highest notification rate in the region at 318.5 per 100,000 population [5]. Maternal Tdap vaccination transfers antibodies across the placenta and protects during this window. The English program reported an effectiveness of 91% (95% CI 84–95) against laboratory-confirmed pertussis in infants younger than 3 months during its early implementation [43]. A United States evaluation of a five-component Tdap vaccine given in pregnancy reported an effectiveness of 96.0% (95% CI 64.6–99.6) against pertussis in the first two months of life and 55.8% (95% CI 27.1–73.2) over the first 12 months, indicating that protection is greatest in early infancy and declines thereafter [44]. The Centers for Disease Control and Prevention recommend a single dose of Tdap in every pregnancy, preferably during weeks 27–36 of gestation [45], and WHO identifies maternal immunization as the additional strategy most likely to be cost-effective for preventing severe disease in young infants [4].

Maternally derived antibodies can attenuate the infant’s own antibody response to the primary series, a phenomenon recognized as blunting. In the Gambian GaPS randomized trial, infants who received a whole-cell primary series and whose mothers had received Tdap-IPV showed significant blunting of pertussis toxin-specific IgG relative to infants of tetanus toxoid-immunized mothers (adjusted geometric mean ratio 0.12, 98.75% CI 0.07–0.22 at 20 weeks and 0.07, 98.75% CI 0.03–0.17 at 9 months), with minimal blunting among infants who received an acellular primary series [18]. The investigators nevertheless reported that antibody quality and memory B-cell responses were preserved regardless of the vaccine given in pregnancy, and that absolute response levels in whole-cell-primed infants remained comparable to or higher than those in acellular-primed infants. Blunting is a measurable immunological effect of unestablished clinical significance: no study has demonstrated that it translates into a higher rate of pertussis in infancy, whereas the benefit of maternal vaccination in the highest-risk period is substantial and consistent across programs. For countries using wP, maternal vaccination should therefore be regarded as complementary to, not competing with, infant priming, and the possibility of blunting is not a reason to withhold it.

## 8. Epidemiological Context in Europe and Poland

### 8.1. The 2023–2024 European Resurgence

Pertussis notifications in the EU/EEA fell to historic lows during the COVID-19 pandemic and then rose steeply. Twenty-nine countries reported 209,674 cases in 2024, of which 86% were classified as confirmed, 4% as probable and 10% as possible. The EU/EEA notification rate was 54.9 per 100,000 population, more than eight times the 2023 rate of 6.7 and 78 times the 2022 rate of 0.7. Four countries—Czechia, Poland, Spain and Germany—accounted for 58% of all reported cases, with Czechia contributing 18% and Poland 15%. Infants under one year of age and individuals aged 10–14 years were the most affected groups, and 53% of cases occurred in people aged 15 years or older. Of 12,827 hospitalized cases with known status, 5246 (41%) were infants under 1 year. Eighty-four deaths were reported, 44 in infants aged one year or under and 29 in people aged 65 years or older [5].

ECDC attributes this resurgence to the expected three-to-five-year epidemic periodicity, the accumulation of unvaccinated and incompletely vaccinated individuals, waning immunity, and reduced natural boosting during the pandemic, compounded by suboptimal coverage of maternal vaccination programs where these exist [46]. The 2024 annual report adds a diagnostic component: 42% of notified cases in 2023–2024 were diagnosed by PCR, compared with 31% in 2020–2022 and 23% in 2015–2019, so part of the measured increase reflects greater use of sensitive molecular testing rather than a proportionate increase in infections [5]. Coverage with three DTP-containing doses among one-year-olds remained high across the region, with a population-weighted EU/EEA average of 92.6% in 2024, and ECDC explicitly notes that surveillance systems and the proportion of laboratory-confirmed cases are heterogeneous, so direct comparisons between countries should be made with caution [5].

### 8.2. The Polish Resurgence and What Explains It

Polish notifications followed the same trajectory as the rest of the region. Reported cases were 753 in 2020, 182 in 2021, 371 in 2022, 922 in 2023 and 32,656 in 2024, corresponding to notification rates of 2.0, 0.5, 1.0, 2.5 and 89.2 per 100,000 population [5]. The 2024 count is therefore 35 times the 2023 count and 88 times the 2022 count. Against the pre-pandemic reference, Poland reported 6828 cases at a rate of 18.0 per 100,000 in its previous epidemic peak year of 2016 [47], so the 2024 rate represents an approximately five-fold increase over the highest level recorded in the preceding decade rather than a return to a familiar peak.

Four observations constrain the interpretation of this increase, and they argue against attributing it to the wP-based priming schedule.

First, the increase was regional rather than Polish. The EU/EEA rate rose more than eight-fold between 2023 and 2024 and 78-fold relative to 2022, and notification rates increased in every reporting country except Croatia and Denmark [5]. Poland’s rate of 89.2 per 100,000 was exceeded by eight EU/EEA countries, all of which use aP for infant priming: Czechia (342.9), Latvia (227.5), Luxembourg (190.6), Norway (183.9), Austria (168.9), Slovakia (134.7), the Netherlands (101.5) and Slovenia (90.4) [5,6]. Czechia, the country with the highest notification rate in the EU/EEA, recorded a rate 3.8-fold that of Poland while using an acellular primary series. This ranking is not an artifact of differing population age structures: the same eight countries exceed Poland on age-standardized rates (Poland 94.7 per 100,000; range 101.2 to 361.2), so the comparison is not explained by Poland having a younger or older population than its comparators [5]. This cross-country pattern argues against a simple explanation based on the Polish priming platform, but ecological surveillance comparisons cannot establish comparative vaccine effectiveness.

Second, Poland’s infant priming performance did not deteriorate during the period in question. WHO/UNICEF estimates of DTP3 coverage among Polish one-year-olds were 94%, 94%, 94%, 95% and 94% in 2020 through 2024 [5]—unchanged across the five years and above the EU/EEA weighted average of 92.6% in 2024. Over the same interval, Czechia’s DTP3 coverage fell from 97% to 86%, an 11% relative decline, and Estonia’s fell by the same relative amount [5]. Stable high coverage makes deterioration in infant priming an unlikely explanation for the Polish increase, whereas declining coverage could have contributed to higher incidence in Czechia.

Third, the age and vaccination-status distribution of European cases locates the susceptibility gap outside the infant primary series. Across the EU/EEA in 2024, 59% of cases aged 10–14 years and 54% of cases aged 15–19 years had received four or more doses, whereas 39% of cases in infants under one year and 18% of those aged 1–4 years were unvaccinated [5]. The burden thus falls both on infants too young or too incompletely vaccinated to be protected and on adolescents whose vaccine-induced protection has waned despite a complete childhood course—the pattern that maternal vaccination and age-appropriate boosters are designed to address, and one that no primary-series platform can resolve on its own.

Fourth, part of the measured Polish increase is likely to be ascertainment. In 2024, 51% of Polish notifications were laboratory-confirmed, compared with 88% in Czechia, 89% in Spain and 97% in Germany [5]. A relatively low confirmed proportion is compatible with a case mix containing more clinically diagnosed and serologically ascertained cases; together with the region-wide expansion of PCR testing, this means that year-on-year notification ratios may not reflect proportional changes in true incidence. Poland’s very low pandemic baseline of 182 cases in 2021 further magnifies relative year-on-year comparisons.

Comparison with two acellular-priming countries is consistent with substantial shared regional drivers but does not establish their relative causal contributions. Czechia, with a population approximately 0.3 times that of Poland, reported 37,375 cases at 342.9 per 100,000 in 2024, a 76-fold increase over 2023, alongside the steepest coverage decline in the region (97% to 86% between 2020 and 2024). Spain reported 26,748 cases at 55.0 per 100,000, an 11-fold increase over 2023, with DTP3 coverage stable at 93–95% throughout. The two countries therefore experienced markedly elevated but quantitatively different notification rates under different coverage patterns: in Czechia declining infant priming coverage may have compounded regional susceptibility, whereas in Spain the increase occurred despite undiminished coverage and is consistent with the same combination of post-pandemic susceptibility accumulation, epidemic periodicity, waning protection in older cohorts and intensified molecular diagnosis that operated across the EU/EEA. ECDC’s own analysis points in this direction: the countries with the highest notification rates were predominantly those in which adolescents aged 10–19 years and adults aged 30 years and above accounted for the largest proportion of laboratory-confirmed cases [5]. Neither the Czech nor the Spanish experience permits a causal attribution to the acellular priming platform, just as the Polish experience does not permit causal attribution to whole-cell priming; the age distributions in all three settings are consistent with an important contribution from waning protection in older cohorts, with declining infant coverage as an additional potential contributor in Czechia.

What can reasonably be concluded is that the Polish rebound is consistent with the region-wide combination of post-pandemic susceptibility, epidemic periodicity, waning adolescent and adult immunity and intensified molecular diagnosis, and that it occurred without any deterioration in infant priming coverage. What cannot be concluded from surveillance data of this kind is a comparative statement about platform performance in either direction: the design does not support attributing the resurgence to wP priming, nor does Poland’s rank below eight aP-using countries constitute evidence that wP priming conferred protection. Answering that question would require product-specific, age-stratified effectiveness studies with standardized case definitions and comparable diagnostic practice.

### 8.3. Where the Susceptibility Lies

Two programmatic priorities follow directly from the age distribution of the burden. Vaccination during pregnancy protects infants in the first weeks of life, before the primary series can take effect. In the EU/EEA, 26 countries had implemented maternal immunization programs, 25 had introduced adolescent boosters and 10 had introduced adult boosters as of April 2026 [5], but where coverage data are available, they indicate suboptimal uptake [46]. Maternal vaccination status, a variable introduced into EU/EEA pertussis surveillance only in 2024, was reported for 1164 cases in infants younger than two years by four of the 29 reporting countries, of which 285 mothers (24%) were recorded as vaccinated during pregnancy—a figure that reflects incomplete data collection as much as it does program coverage and illustrates how weakly this intervention is currently monitored [5]. Adolescent and adult boosters address the second susceptibility pool, and the 25-versus-10-country contrast indicates that adult booster recommendations remain the least widely adopted of the three strategies. Both interventions rely exclusively on aP-containing products, so their expansion is compatible with, rather than an alternative to, wP-based infant priming.

## 9. Vaccine Confidence, Acceptance and Communication

Reactogenicity influences vaccine acceptance. Whole-cell vaccines cause more fever and local reactions, which can lead caregivers to hesitate over subsequent doses, and historical controversies about wP safety contributed to marked declines in coverage and to epidemics in several countries during the 1970s and 1980s [48]. Contemporary vaccine hesitancy is a multifactorial phenomenon driven by risk perception, trust, the information environment, and access, with reactogenicity as one of several contributing factors [49].

Polish monitoring has documented a long-term rise in recorded instances of non-compliance with mandatory childhood vaccination, from 16,689 in 2015 to 87,344 in 2023 [50]. These records should not be read directly as a count of unique, permanently unvaccinated children, because they can also capture delayed vaccination and other administrative circumstances [50]. The trend nonetheless indicates that parental acceptance and sustained uptake matter at least as much for program performance as the choice of vaccine technology [3,49].

In Poland, both platforms coexist and privately purchased highly combined aP-containing vaccines have historically accounted for a substantial proportion of infant vaccination. A 2015 Supreme Audit Office report found that approximately 60% of children up to 18 months of age were vaccinated with privately financed highly combined vaccines [51,52]. Paradowska-Stankiewicz subsequently analyzed Polish surveillance data from 2012–2016 using alternative assumptions regarding platform use, including an aP:wP distribution of 65:35 derived from demographic and procurement/sales data [51]. These figures describe a historical period and should not be extrapolated to current platform uptake without contemporary national data.

Communication implications are practical. Explaining that a higher rate of fever and local reactions is not evidence of a higher risk of serious harm, that acellular products are available for defined clinical indications, and that completing the schedule on time matters more than the choice between platforms is likely to be more effective than platform advocacy in either direction.

## 10. Supply, Cost and Program Resilience

Several EU/EEA countries experienced constrained access to aP-containing vaccines during supply shortages in the mid-2010s, requiring temporary prioritization of doses [53]. A national program dependent on a single platform and a small number of suppliers may be more vulnerable to such disruption; diversified procurement and contingency access are therefore relevant to program resilience. Poland currently has access to a domestically produced wP-containing DTP vaccine alongside multiple aP-containing products [54,55]. The national vaccination portal currently lists one wP-containing DTP product, seven pediatric aP-containing products (including 4-in-1, 5-in-1, and 6-in-1 formulations), and four Tdap/Tdap-IPV products; Tripacel is listed as unavailable [54]. Under the national immunization program, DTwP is publicly funded for routine infant priming, while aP-containing products are used for specified indications and may also be purchased privately [7,54]. Pertussis vaccination during pregnancy is publicly funded in primary care at 27–36 weeks of gestation and, when preterm delivery is considered likely, may be given after 20 weeks [56]. Adults can receive pertussis-containing vaccines in pharmacies participating in the National Health Fund (NFZ) vaccination program; the NFZ covers pharmacist qualification and vaccine administration, while the cost of the vaccine itself depends on reimbursement eligibility [57].

## 11. Programmatic Implications

The evidence reviewed here does not support a claim that either platform is clinically superior across all outcomes and settings. It yields a more specific set of conclusions with direct programmatic consequences, summarized in Table 2.

## 12. Strengths and Limitations

The principal strength of this review is that it treats the platform comparison as a multidimensional question, combining immunological, clinical, safety, epidemiological, behavioral and supply evidence, and that it distinguishes product-level from platform-level statements throughout. It also grounds the programmatic discussion in a single, unusually informative national example.

The limitations are substantial and constrain the conclusions. The review is narrative: the search was purposive rather than systematic; no protocol was registered; no risk-of-bias assessment was performed; and selection bias cannot be excluded. Much of the decisive efficacy evidence comes from trials conducted in the 1990s with products, schedules, and case definitions that differ from those currently in use, and it cannot be assumed that a contemporary wP or aP product would reproduce those estimates. The comparative effectiveness studies of wP- versus aP-primed cohorts are observational and subject to confounding by birth cohort, calendar time, diagnostic practice and secular changes in circulating strains. No validated correlate of protection exists, so serological comparisons cannot be translated into clinical protection, and the seroepidemiological durability estimates cited here are serological endpoints only. Surveillance comparisons between countries are affected by heterogeneous case definitions, testing intensity, and confirmation practices, as ECDC itself emphasizes, and the Polish year-on-year ratios reported above should therefore not be interpreted as direct measurements of changes in true incidence. Mechanistic studies of tissue-resident memory and antibody subclass involve small, selected samples. Finally, the review addresses one national program in detail, and its programmatic conclusions are not directly transferable to settings with different coverage, schedules, surveillance systems or supply arrangements.

## 13. Conclusions

Whole-cell and acellular pertussis vaccines differ consistently in two respects. Whole-cell priming establishes a Th1/Th17-oriented cellular profile that can persist into adolescence and is associated with an IgG1-dominant antibody response. In contrast, acellular vaccination produces higher and higher-avidity antibody concentrations with a rising IgG4 fraction and is substantially better tolerated. On clinical endpoints, however, no platform is uniformly superior across products, schedules, and outcomes. Historical placebo-controlled trials showed substantial product-level heterogeneity, with efficacy estimates of 36–48% for two poorly performing whole-cell vaccines and 84–85% for multicomponent acellular vaccines; a United Kingdom whole-cell product performed comparably to a five-component acellular vaccine. Protection wanes after acellular priming and boosting, and observational comparisons favor whole-cell-primed cohorts, though these comparisons involve historical products under changing diagnostic conditions. Neither intramuscular platform reliably prevents colonization or transmission. Higher reactogenicity of whole-cell vaccines is well established; a causal relationship with permanent neurological injury is not.

For a whole-cell-priming program such as Poland’s, the evidence reviewed here does not establish that a platform change would improve clinical or programmatic outcomes, and the 2024 resurgence does not by itself provide evidence that such a change is warranted: it was region-wide and occurred while Polish infant coverage remained unchanged at 94%, with higher notification rates reported in eight acellular-priming countries. Neither does it establish that whole-cell priming conferred protection, since surveillance data cannot support comparative platform claims in either direction. Key determinants of program performance include timeliness and completeness of the infant series, maternal vaccination in every pregnancy, age-appropriate boosters for adolescents and adults, transparent communication that separates reactogenicity from serious harm, and secure access to both platforms. Whether contemporary whole-cell products confer clinically meaningful advantages over contemporary acellular products under current epidemiological conditions remains an open question that requires product-specific, age-stratified effectiveness studies with standardized case definitions. Mucosal vaccine candidates addressing the colonization gap common to both platforms are in early clinical development and are not yet relevant to policy.

## Figures and Tables

**Table 1:** Comparison of whole-cell (wP) and acellular (aP) pertussis vaccine platforms.

Domain	Whole-Cell (wP)	Acellular (aP)	Interpretation
Antigenic composition	Killed whole *Bordetella pertussis*; broad antigen repertoire and intrinsic innate stimuli	Defined purified antigens (pertussis toxin ± filamentous hemagglutinin, pertactin, fimbriae), alum-adsorbed	Antigen breadth differs; neither composition has a validated correlate of protection
T-helper polarization	Th1/Th17-oriented; profile persists into adolescence despite aP boosters	More Th2-skewed	Consistent immunological difference; not a validated predictor of clinical protection
Antibody quantity and avidity	Lower antibody concentrations after preschool boosting than aP-primed children	Higher concentrations and higher avidity indices; more readily boosted	Quantitative advantage for aP priming that does not translate into demonstrated clinical superiority
Antibody subclass profile	IgG1-dominant; efficient complement fixation and FcγR engagement	Progressively greater IgG4 fraction with repeated exposure; IgG4 does not fix complement and binds Fcγ receptors weakly	Reduced effector capacity per unit of antigen-specific IgG is mechanistically plausible; clinical significance not established
Efficacy in placebo-controlled trials	Highly product-dependent (36–48% for two poorly performing products; a UK product matched a five-component aP vaccine)	Product-dependent (84–85% for multicomponent products; 59–78% for one- and two-component products)	Product identity matters more than platform label
Durability	Several years; longer-lasting cellular imprint; lower adolescent disease risk in wP-primed cohorts	Wanes within a few years after the fifth dose and after Tdap boosting	Neither platform provides lifelong protection
Reactogenicity	Higher rates of fever, local reactions, irritability and protracted crying	Substantially better tolerated; comparable to diphtheria–tetanus control in placebo-controlled trials	Consistent and clinically relevant difference
Serious adverse events	No established causal link with permanent encephalopathy	Favorable safety profile	Reactogenicity must not be equated with serious harm
Colonization and transmission	Shortened colonization but not sterilizing in a non-human primate model	Protects against disease but does not prevent infection or transmission in the same model	Direct human transmission evidence is limited for both platforms
Age and indication	Infant and early childhood priming, within product age limits	Infant priming; age-appropriate boosters in older children, adolescents, and adults; vaccination during pregnancy; all within product-specific indications	Access to aP is indispensable even in a wP-based program
Manufacturing and supply	Established global platform; product heterogeneity requires strong quality control	More complex antigen-specific production; combination-product supply can be constrained	Diversified procurement and contingency planning are required

Abbreviations: aP, acellular pertussis vaccine; FcγR, Fc gamma receptor; IgG, immunoglobulin G; Th, T helper; UK, United Kingdom; wP, whole-cell pertussis vaccine.

**Table 2:** Programmatic consequences for a whole-cell-priming national program.

Domain	Evidence Position	Programmatic Implication
Platform choice	No uniform clinical superiority of either platform across products, schedules, and outcomes; efficacy is product-specific	A change in platform is not justified by mechanistic differences alone; any change would require product-specific effectiveness evidence and an assured booster and maternal strategy
Timeliness and completeness	Infant risk is concentrated before the primary series is complete	Prioritize an on-time first dose and full series completion over the platform debate
Maternal vaccination	Consistent, substantial effectiveness in the highest-risk period; blunting is measurable but of unproven clinical significance; monitoring is weak across the EU/EEA	Vaccinate in every pregnancy; do not withhold on account of blunting; record and report maternal vaccination status
Boosters	Protection after aP priming and Tdap boosting wanes within a few years; adolescents aged 10–14 years are among the most affected groups	Maintain age-appropriate boosters and monitor their timing and uptake
Reactogenicity	Consistently higher with wP; no established causal link to permanent neurological injury	Communicate the distinction explicitly; support families through expected reactions
Surveillance and research	Country-level “wP versus aP” comparisons are confounded by case definitions, testing intensity and confirmation practice	Invest in product-specific, age-stratified effectiveness studies and in raising the laboratory-confirmed proportion of notifications
Supply	Combination-product supply has been constrained in the EU/EEA within the past decade	Diversify procurement and maintain contingency access to both platforms

Abbreviations: aP, acellular pertussis vaccine; EU/EEA, European Union/European Economic Area; wP, whole-cell pertussis vaccine.

## Data Availability

No new data were created or analyzed in this study. All information discussed is derived from the cited publications and official documents; the surveillance figures reported in Section 8 are available from the cited European Centre for Disease Prevention and Control reports.

## Abbreviations

The following abbreviations are used in this manuscript:

aP	acellular pertussis vaccine
CI	confidence interval
DTaP	diphtheria, tetanus and acellular pertussis vaccine (pediatric, full-dose toxoids)
DTP3	third dose of diphtheria–tetanus–pertussis-containing vaccine
DTwP	diphtheria, tetanus and whole-cell pertussis vaccine
ECDC	European Centre for Disease Prevention and Control
EU/EEA	European Union/European Economic Area
IgG	immunoglobulin G
IPV	inactivated poliovirus vaccine
PCR	polymerase chain reaction
Tdap	tetanus, reduced-antigen-content diphtheria and reduced-antigen-content acellular pertussis vaccine
Th	T helper
wP	whole-cell pertussis vaccine
WHO	World Health Organization
